# PP95 Conceptual Mapping Of Health-Related Quality Of Life, Quality Of Life, And Well-Being: A Systematic Review

**DOI:** 10.1017/S0266462325103127

**Published:** 2025-12-29

**Authors:** Fernando Argento, Federico Augustovski, Maria Belizan, Natali Ini

## Abstract

**Introduction:**

Assessing health-related quality of life (HRQoL), quality of life (QoL), and well-being (WB) are essential for measuring health outcomes. This study provided a conceptual mapping of these measures, proposed evidence-based frameworks, and linked them to widely used generic patient-reported outcome measures.

**Methods:**

A systematic literature review was conducted that included articles from databases and gray literature through to December 2023. Studies describing HRQoL, QoL, and WB concepts with their dimensions or domains were included, focusing on general population studies without design restrictions. Domains, subdomains, and facets were extracted and analyzed based on frequency and qualitative methods. These frameworks were then mapped to commonly used generic patient-reported outcome measures.

**Results:**

From 14,114 papers, 35 studies and 36 frameworks were included (HRQol=15, Qol=5, WB=16). A total of 168 first, 225 second, and 79 third-level domains were retrieved and curated into 118 unique entities: HRQoL had 82 dimensions, QoL had 49, and WB had 69. Overlap analysis revealed 26 shared dimensions, with 26 unique to WB, 29 to HRQoL, and seven to QoL. Final models included 18 HRQoL, 22 QoL (18 from HRQoL plus 4), and 10 WB dimensions. Mapping of patient-reported outcome measures showed coverage for HRQoL of 59.4 percent, QoL of 65.9 percent, and WB of 34 percent. The EuroQol Health and Wellbeing instrument had the broadest coverage for HRQoL (17/18) and WB (5/10).

**Conclusions:**

Significant inconsistencies and overlaps exist among definitions and domains for HRQoL, QoL, and WB. The lack of clear conceptual and operational definitions hinders the validity of measurement tools. This study underscored the need for clearer definitions and provided an initial step toward better understanding and measurement of these essential health and policy concepts.

